# Statistical field theory of the transmission of nerve impulses

**DOI:** 10.1186/s12976-020-00132-9

**Published:** 2021-01-06

**Authors:** Gianluigi Zangari del Balzo

**Affiliations:** Sapienza Università di Roma, Rome, Italy

**Keywords:** Action potentials, Ion channels, Hodgkin-Huxley model, Ising model, Statistical field theory, Quasi particles, Multiple sclerosis, Particle physics, Complex networks

## Abstract

**Background:**

Stochastic processes leading voltage-gated ion channel dynamics on the nerve cell membrane are a sufficient condition to describe membrane conductance through statistical mechanics of disordered and complex systems.

**Results:**

Voltage-gated ion channels in the nerve cell membrane are described by the Ising model. Stochastic circuit elements called “Ising Neural Machines” are introduced. Action potentials are described as quasi-particles of a statistical field theory for the Ising system.

**Conclusions:**

The particle description of action potentials is a new point of view and a powerful tool to describe the generation and propagation of nerve impulses, especially when classical electrophysiological models break down.

The particle description of action potentials allows us to develop a new generation of devices to study neurodegenerative and demyelinating diseases as Multiple Sclerosis and Alzheimer’s disease, even integrated by connectomes. It is also suitable for the study of complex networks, quantum computing, artificial intelligence, machine and deep learning, cryptography, ultra-fast lines for entanglement experiments and many other applications of medical, physical and engineering interest.

## Background

**Fig. 1:**
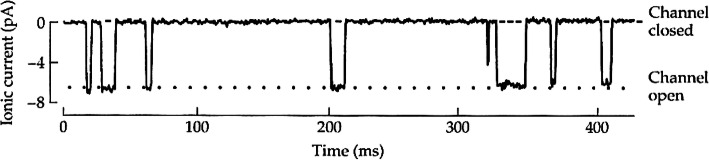
The flow of an ionic current of the order of 6.6 pA through a small axonal membrane element shows eight ion channel openings (corresponding to a flow of 4.1 × 10^7^ ions per second through a single pore). (Courtesy of B. Hille, [5])

In 1952 British physiologists Sir Alan Lloyd Hodgkin (1914–1998) and Sir Andrew Fielding Huxley (1917–2012) at the University of Cambridge demonstrated the existence of selective and voltage-dependent ion channels in the nerve cell membrane with five famous pioneers works published in the Journal of Physiology. They received the Nobel Prize for Medicine in 1963 together with the Australian physiologist Sir John Carew Eccles (1903–1997) [1–5]. Nowadays, after almost 68 years, the success and evolution of the Hodgkin and Huxley models, hereinafter referred to simply as” HH models”, are still alive and continuously stimulate the development of new topics and branches of physiology and neurosciences [5–8]. In 1976, the “Patch Clamp” method, developed by Erwin Neher and Bert Sakmann [9], who received the Nobel Prize in Medicine in 1991, demonstrated among other things:

**1. Microcurrents**. The existence of microscopic electric currents of intensity in the order of pA (picoampere) that flow through each ion channel, transporting on average thousands of ions per millisecond (Fig. 1);

**2. Stochastic channels**. The stochastic dynamics of opening/closing (“gating”) of each ion channel.

## Methods

The recording of current flow through individual channels (Fig. 1), shows stochastic fluctuations between closed and open states. This is a sufficient condition to define the concept of membrane conductance through statistical mechanics of disordered and complex systems [10–15]. We will therefore start from the basic formalism developed by Hodgkin and Huxley to describe the processes carried out by the conductances of the Na^+^ and K^+^ channels to explain the generation of action potentials. The opening and closing of voltage-gated ion channels (“gating”) is a physical process that involves complex conformational changes in the structure of each channel, or in the sub-units of which it is composed. Opening a gate is generally called “conductance activation”, while closing “conductance deactivation” [5–8].

### The Ising model

**Fig. 2:**
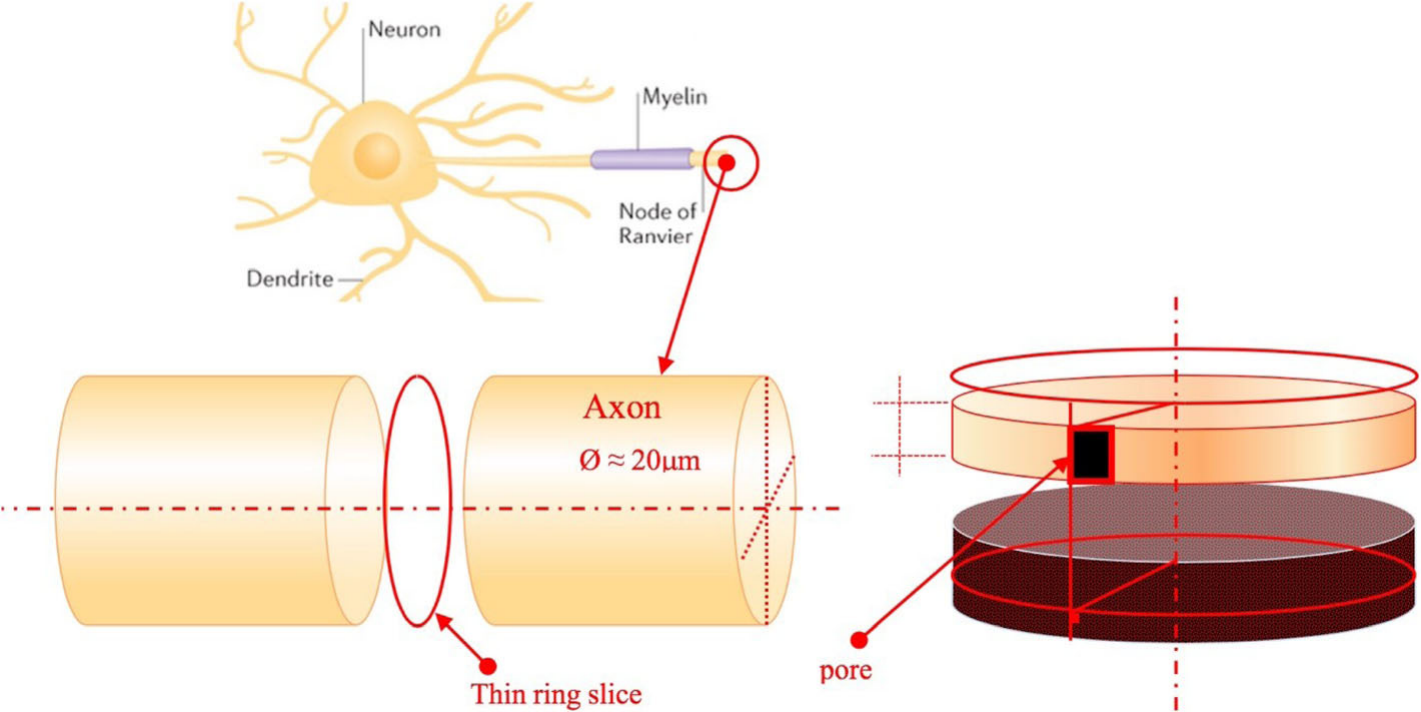
N voltage-dependent ion channels on an elementary region (slice) of an axon made by a thin ring of radius *ρ * ≈10 μm and thickness h ≈1 nm

**Fig. 3:**
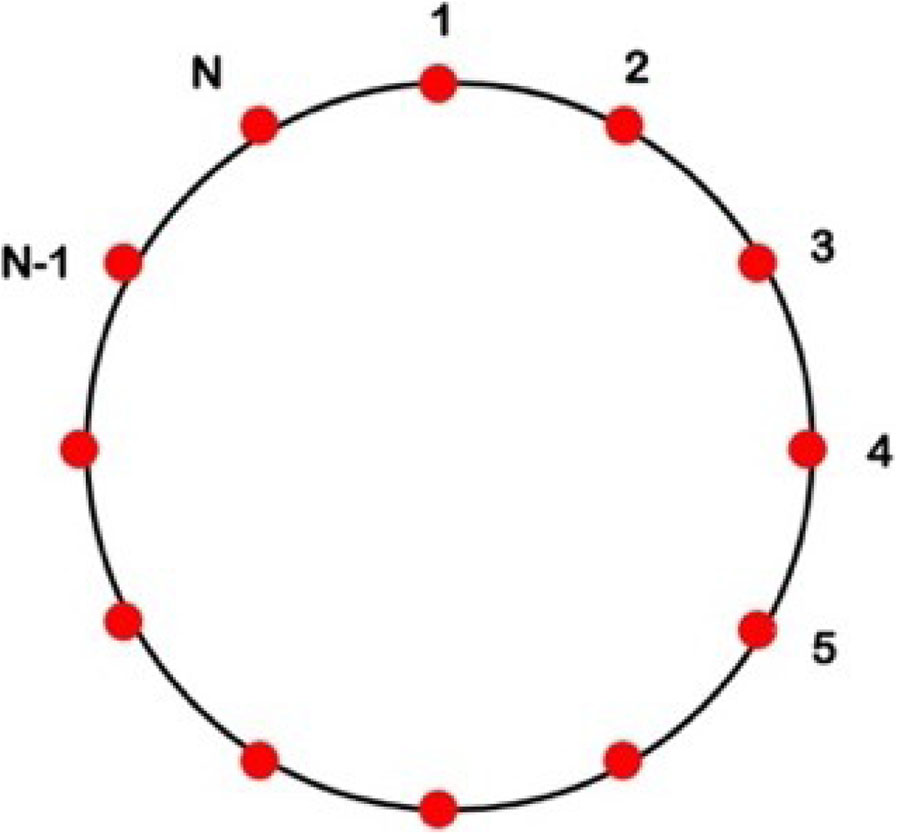
Topology of the one-dimensional Ising model, after K. Huang [13]

We define gating as an Ising spin variable. We will therefore consider a distribution of N voltage-dependent ion channels on an elementary region (slice) of a nerve membrane (axon) made by a thin ring of radius ρ ≈ 10 μm and thickness h  ≈1 nm. (See Fig. 2). A population of N ion channels of a certain superfamily (Na^+^, K^+^, Cl^−^,..) will be distributed on an axon section made by a thin ring (represented in Fig. 2 and topologically modeled in Fig. 3), formally described by the Hamiltonian of the one dimensional Ising Model for each superfamily of channels:

1 $$ {H}_I=-J{\sum}_{i=1}^N{S}_i{S}_{i+1}-\phi {\sum}_{i=1}^N{S}_i $$

The border condition (See Fig. 3) is S_N + 1_ = S_1_, where S_i_ are N Ising variables (S_i_ = +1 corresponds to an open channel state, while S_i_ = − 1 corresponds to a closed channel state, for i = 1, ….,N). The energy of interaction between the channels of the same superfamily is represented by the variable J > 0, which we assume isotropic and” ferromagnetic”, while *ϕ* (mV) is the electrochemical driving force, hereinafter called “driving force” *ϕ* = V - E_γ_ where V (mV) is the membrane potential and E_γ_ (mV) is the equilibrium potential of each superfamily of ion channels.

The Helmholtz Free Energy will be [13].

2 $$ {A}_I\left(\phi, \beta \right)=- NJ-\frac{N}{\beta}\mathit{\log}\left[\cosh \left(\beta \phi \right)+\sqrt{{\mathit{\sinh}}^2\left(\beta \phi \right)+{e}^{-4\beta J}}\right] $$

And the magnetization will be

3 $$ {M}_I\left(\phi, \beta \right)=\left\langle {\sum}_{i=1}^N{S}_i\right\rangle =-\frac{\partial }{\partial \phi}\left[{A}_I\left(\phi, \beta \right)\right]=\frac{Nsinh\left(\beta \phi \right)}{\sqrt{{\mathit{\sinh}}^2\left(\beta \phi \right)+{e}^{-4\beta J}}} $$

Where $\beta =\frac{1}{k_BT}$; k_B_ is the Boltzmann constant and T the absolute temperature. We will see shortly what the observables of the Ising model mean in our case, above all the magnetization, which will be our main observable. Before that it is necessary to discuss a formal issue about the relationship between our stochastic model and the Hodgkin-Huxley model, which will help us to explain our choices.

### Ising and Hodgkin-Huxley

**Fig. 4:**
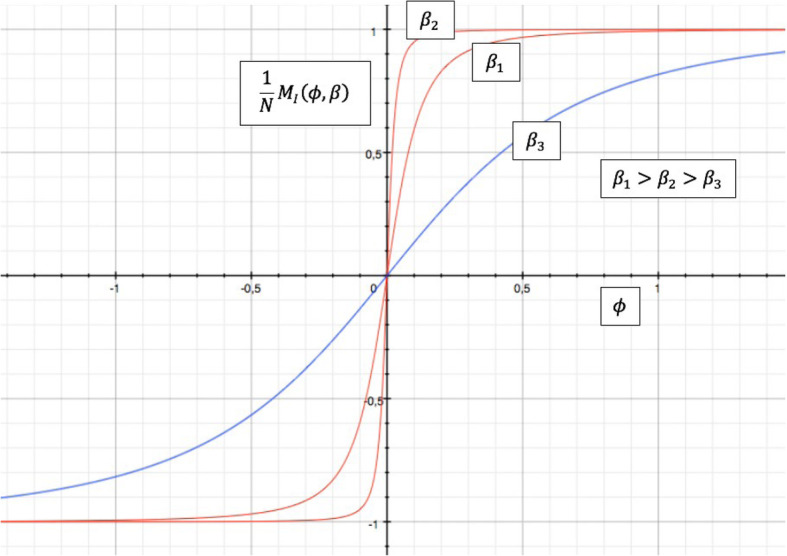
Spin magnetization in the one-dimensional Ising Model for three different “temperatures” and J = 1

**Fig. 5:**
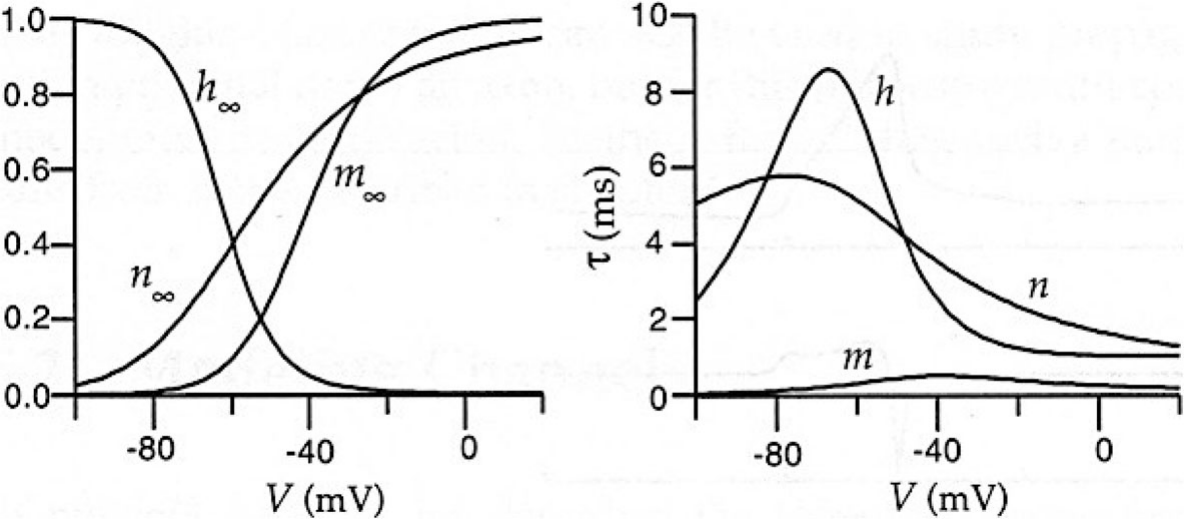
Characteristic limit ratios as a function of membrane potential in the HH model. The figure on the left shows the limit functions for activating the K^+^ conductance (n_*∞*_) and the activation and inactivation functions for the Na^+^ conductance (m_*∞*_, h_*∞*_). The relative time constants (as a function of potential) are shown on the right. (courtesy of P. Dayan et Al., [8])

**Fig. 6:**
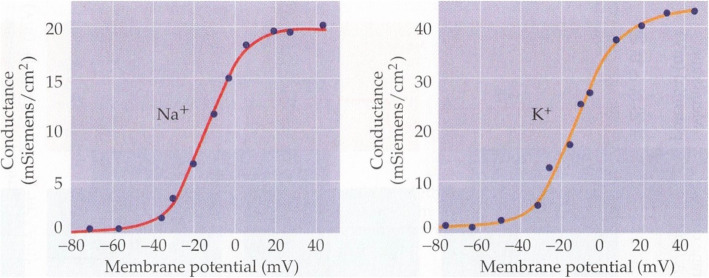
Conductances as a function of the membrane potential for the Na^+^(left) and K^+^(right) channels. (Courtesy of Purves [6], after Hodgkin and Huxley [2])

The sigmoid distribution of spin magnetization reproduced in Fig. 4 recalls the activation and inactivation limit functions n_∞_, m_∞_, h_∞_ defined by the HH models (Fig. 5) and the conductances in function of the membrane potential for the Na^+^ and K^+^ channels (Fig. 6). The sigmoid characteristic is typical of a cooperative process, as in the present case. In the HH model, the limit function for the conductance is

4 $$ {n}_{\infty}\left(\phi \right)=\frac{\alpha_n\left(\phi \right)}{\alpha_n\left(\phi \right)+{\beta}_n\left(\phi \right)} $$

expressed by the gating fractions α_n_(V) and β_n_(V) as a function of the potential [5, 7, 8].

The gating fractions are defined on the basis of general thermodynamic considerations (Boltzmann) [5, 7, 8].

5 $$ {n}_{\infty }(V)=\frac{1}{1+\left(\frac{A_2}{A_1}\right)\exp \left(\frac{\left({B}_1-{B}_2\right)\phi }{\phi_T}\right)} $$

In the present case, the choice of the one-dimensional Ising model obeys a different methodological choice that we use to call “congruence” because it wants to express a “special” link between the physics of gating process and its mathematical law in a closed form, that is without using a “metatheory”. Therefore, to interpolate the experimental data (Fig. 6), we discard function (5) of the HH model because it is a “metatheory”, but we choose the one-dimensional Ising model because it presents the congruence in closed form we are looking for.^1^

## Results

### The Ising conductance

Recall the expression (3) for the magnetization in the one-dimensional Ising model. According to our methodological constraint, here the magnetization becomes the conductance of the nerve membrane.

We thus define the “Ising conductance” g_I_ as the magnetization:

6 $$ {g}_I:= {M}_I=\left\langle {\sum}_{i=1}^N{S}_i\right\rangle $$

In practice, we will consider the specific conductance (mSiemens / cm^2^) so that the membrane current per unit area will be expressed by Ohm’s Law

7 $$ {i}_m={g}_I\left(V-{E}_{\gamma}\right) $$

Where V (mV) is the membrane potential and Eγ (mV) is the equilibrium potential of each superfamily of channels.

**Fig. 7:**
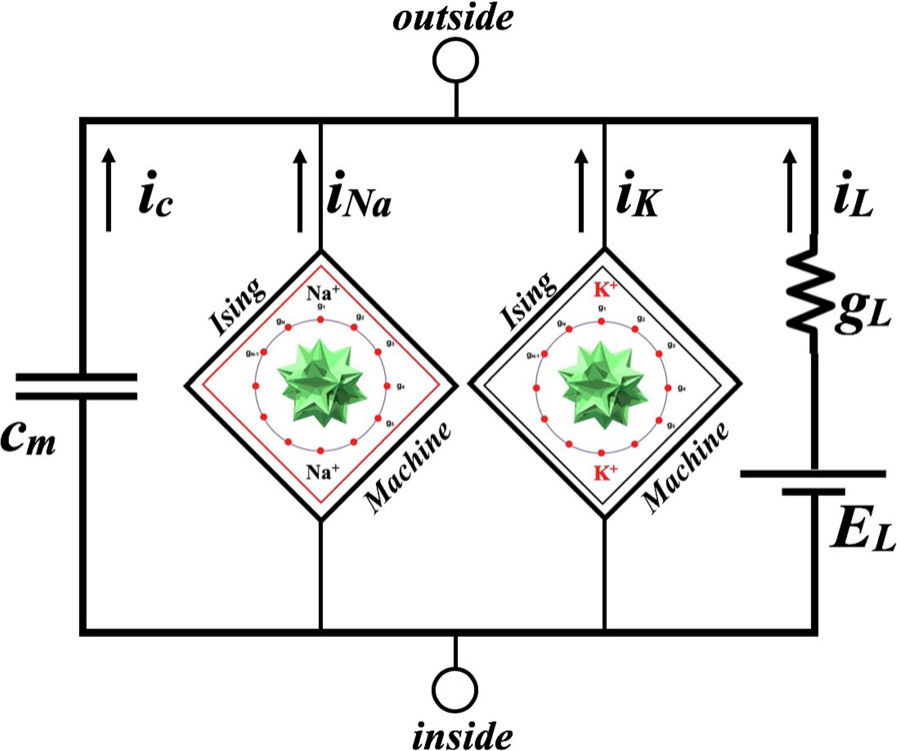
The “Ising Neural Machine”. The equivalent single compartment circuit containing two stochastic elements called “Ising Neural Machines”, respectively for the Na^+^channels and for the K^+^channel s[1]. By “inside” and “outside” is meant inside and outside the nerve cell membrane. The specific capacity of the membrane is indicated as c_*m*_ while i_*L*_, g_*L*_ and E_*L*_ indicate respectively the “leakage current ”[2] per unit area, the leakage conductance and potential. The currents leaving the two Ising machines are total currents (per unit area)

From our model we define a stochastic circuit element which we call for convenience of reading “Ising Neural Machine” (INM), briefly “Ising Machine” (which we abbreviate as “Ising N-Machines”, “INMs” or just “Ising Machines”) and we indicate it with a rhomboid frame icon.^2^ We place the INMs in the equivalent circuit with single compartment of Fig. 7 defined for two superfamilies of ion channels (Na^+^ and K^+^).

Now we want to discuss the problem of the generation of action potentials. In the following we will for brevity refer to action potentials as *spikes*.

### Nuons

**Fig. 8:**
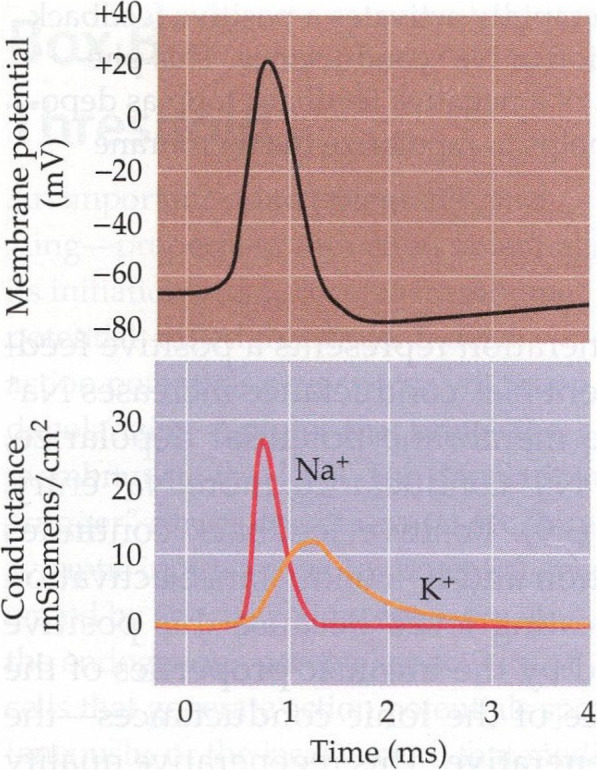
Reconstruction of an action potential after Hodgkin and Huxley, 1952d [4], courtesy of Purves [6]

With reference to Fig. 8 [6], which shows the reconstruction of an action potential after Hodgkin and Huxley, 1952d [4, 6], we find that an increase in the conductance of the Na^+^ channels triggers a spike. A flow of Na^+^ ions enters the nerve cell, causing the membrane potential to depolarize up to the E_Na_ value. The depolarization activates the (delayed) conductance of the K^+^ channels which provokes the escape of K^+^ ions from the nerve cell, thus blocking the Na^+^ channels and repolarizing the membrane up to the E_K_ value (“refractory period”). Since K^+^ conductance becomes transiently higher than its rest value, the membrane potential exceeds its negative rest value (“hyperpolarization”), so that both the K^+^ conductance and the (possibly) residual Na^+^ conductance are inactivated. Finally, the membrane returns to its resting value and it is ready to trigger a new spike.

**Fig. 9:**
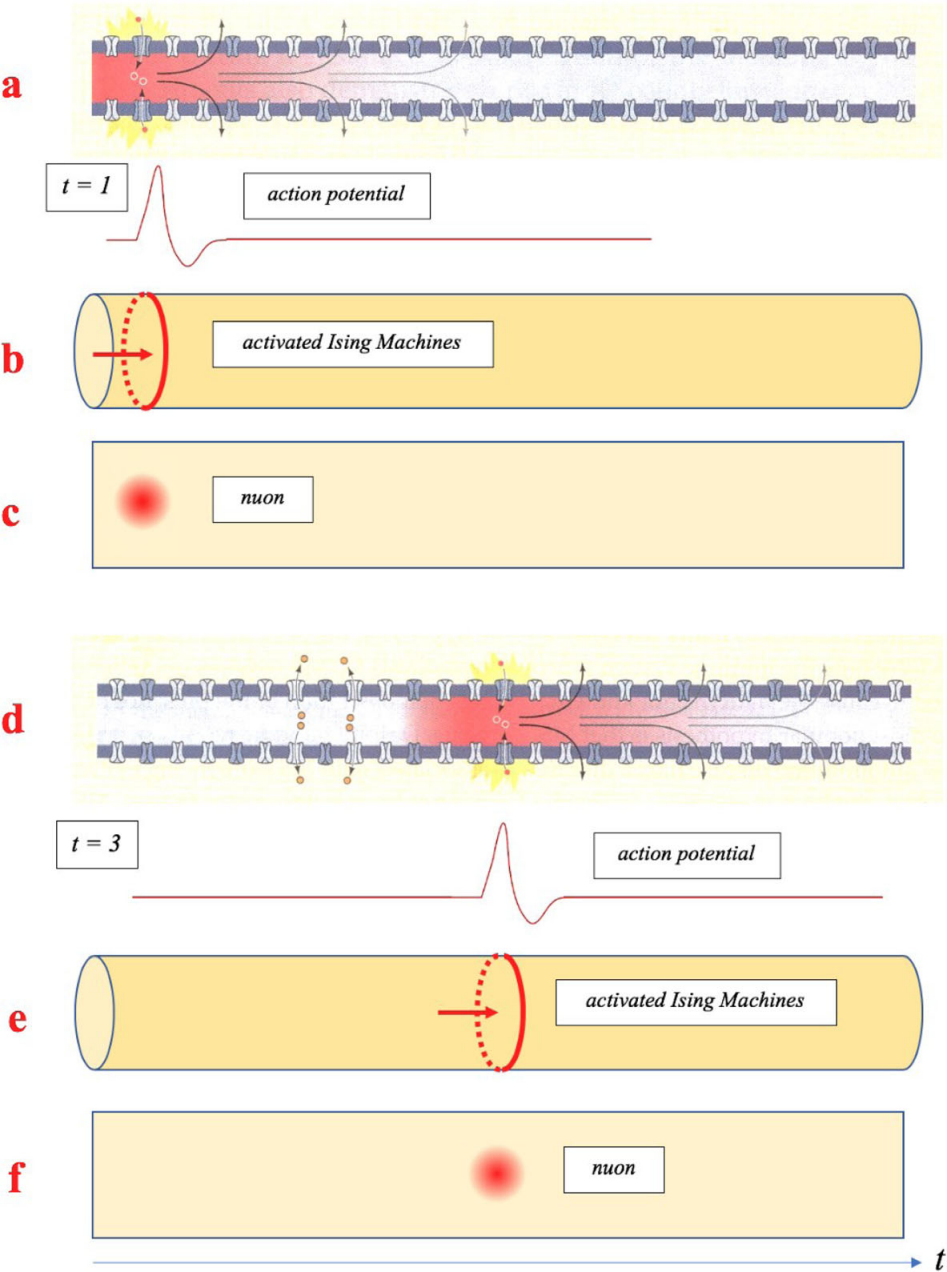
Spikes and nuons. **a** and **d** (modified after D. Purves, [6]), show the motion of a spike for t = 1 and t = 3 (arbitrary units). **b** and **e** show the trigger of a pair of Na^+^/K^+^ Ising machines housed in annular regions of the membrane for t = 1 and t = 3 (arbitrary units). **c** and **f** show the motion of a nuon along the axon for t = 1 and t = 3 (arbitrary units)

With reference to the next Fig. 9, the local depolarization of the nerve membrane is started by a current of carriers (i.e. a synaptic potential, an artificial stimulus, or a passive current) which triggers a spike (see Fig. 9a and d). From our point of view, consider for a certain instant t > 0 a single carrier triggering a pair of “Ising machines” (Na^+^/K^+^) housed into an annular section of the nerve cell membrane (See Figs. 2, 9b and e). As it will be clarified below, only one annular section is activated at a time t > 0.

At this point, we remind that the activation of the voltage-dependent ion channels involves reversible conformational changes in the membrane of the nerve cell (gating) which configure a structural deformation of the membrane itself. The deformation tends to chase the carrier and to propagate along the nerve axis in the direction of the carrier itself, activating one annular section at a time. The refractory period prevents the spike from propagating backwards and, at the same time, stops the generation of further spikes, in order to fire one spike at a time.

This phenomenon shows strong similarities with the concept of *polaron* by H. Fröhlich [22–27] which describes an electron that moves with its field of deformation (see also RP Feynman, 1954, [28, 29]). In that case, the carrier together with the induced deformation can be considered as one entity: a *quasi-particle* called *polaron*.

In the present case, following H. Fröhlich’s concept of *polaron*, we define the spike wave function *Ψ*_spike_ by exploiting the formalism of the “*Produkt-Ansatz*” by L. D. Landau (1933) [30] as the following (in kets):

8 $$ \left|{\Psi}_{spike}>=\left|\varphi {\left(\boldsymbol{r}\right)}_{carrier}\right\rangle \right|\left. field\right\rangle := \left|\varphi \left(\boldsymbol{r}\right)\left.{}_{carrier}\right\rangle \right|\left. Ising\right\rangle $$

Where | *φ*(***r***)_*carrier*_⟩ is the carrier wave function while ∣*field*⟩ is the field of the Ising Machine and **r** is the position operator of the carrier along the axon axis in the direction of propagation of the carrier.

The total Fröhlich Hamiltonian function of our model will be given by:

9 $$ H={H}_{Carrier}+{H}_{Ising}+{H}_{Carrier\divideontimes Ising}=\frac{{\boldsymbol{p}}^2}{2{m}_c}+{H}_{Ising}+{H}_{Carrier\divideontimes Ising} $$

Where **p** is the canonically conjugate momentum operator of the carrier of mass m_c_ and H_Ising_ the Hamiltonian function (1). In this way, we can interpret the spike as a quasi- particle which represents the carrier together with the induced deformation on the nerve cell membrane.

We call this quasi-particle *nuon* and denote it with the letter ñ. The statistical field theory that foresees the concept of nuon will for brevity be called SFT [ñ].

As a first approximation, if we consider the density of ion channels on the axonal membrane of non-myelinated axons almost constant [6, 31–34], we can neglect the composition terms H_Carrier*Ising_ in (9) after the trigger of the first spike, because the process is ruled only by the Ising machines.

Therefore, we consider the process of generation and transmission of a spike for a time t > τ, where τ is the generation time of each spike by each pair (Na/K) of Ising Machines. If we indicate with s the coordinate along with the axis of the axon (which coincides with the axis of the coaxial annular sections), then each section (housing a pair of Ising Machines Na/K) will be traveled in a time t by the coordinate s = s (t) Therefore, the velocity v = ds/dt of the nuon will be given by the limit of the difference quotient ∆s/∆t with ∆t ≠ 0. Our choice of the one-dimensional Ising model is thus clear. Finally, we will have

10 $$ H\approx \frac{{\boldsymbol{p}}^2}{2{m}_c}+{H}_{Ising} $$

## Discussion

### The saltatory conduction

**Fig. 10:**
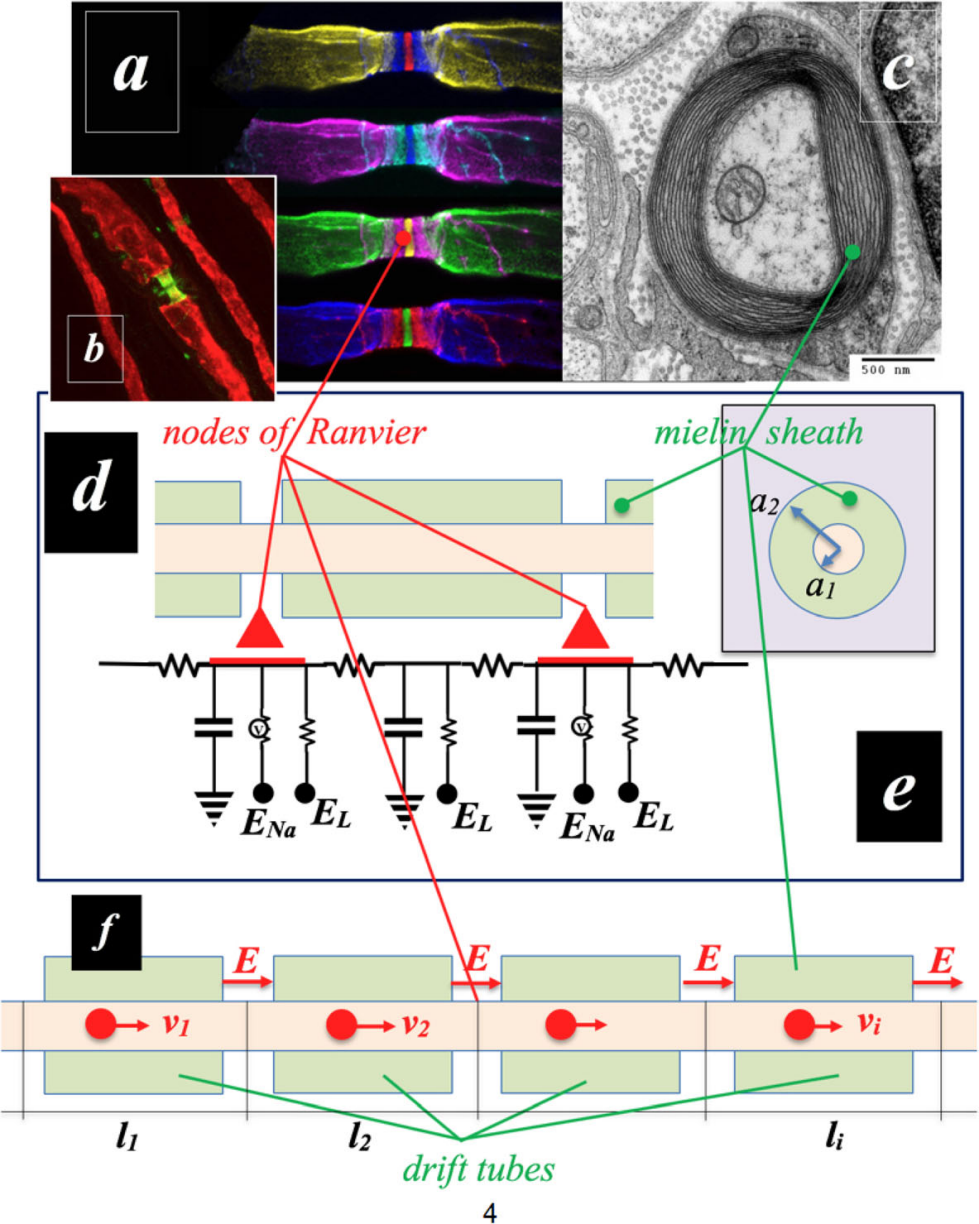
Nodes of Ranvier and Myelin Sheath. **a-**Courtesy of Anne Desmazieres et. Al. [36]. **b-**Courtesy of Prof. Peter Brophy [37]. **c**-Transmission electron micrograph of a myelinated axon. The myelin layer (concentric) surrounds the axon of a neuron, showing cytoplasmathic organs inside [38]. **d**, **e**-The nodes of Ranvier and the myelinated regions of an axon are represented as an equivalent circuit within a intercompartmental model, modified after F, Dayan et al., [8]. **f**-Schematic of our model a μLINAC, where an electric field acts in the nodes of Ranvier and accelerates the nuons in the myelinated sections that behave like drift tubes

An application case of neurological interest is that of the so-called “saltatory conduction” in myelinated axons. Measures of the average velocity of spikes in non-myelinated axons are between 0, 5 and 10 m/s, while in myelinated axons are up to circa 150 m/s [6, 31–34]. Multiple Sclerosis (MS) is a serious pathology of the central nervous system (CNS) characterized by a complex of clinical disorders caused by the bad conduction of spikes, as a consequence of damage and/or total or partial loss of the myelin sheath (demyelination) due to the inflammation of the axon pathways [6, 31–35]. The study of saltatory conduction is therefore crucial to understand and deal with these serious diseases. Saltatory conduction is described by means of the “cable theory”. Let’s now see how some relations derived from cable theory can be interpreted in the context of SFT [ñ]. We can model the myelin sheath as composed of a series of concentric thin cylindrical surfaces of length L, capacity per unit of area c_m_ and thickness d_m_ distributed from the radius a_1_ of the axon core to the external radius a_2_, that is to the axon radius (See next Fig. 10).

We will then have a total capacity C_m_ (series) given by the following relations [8]

11 $$ \frac{1}{C_m}=\frac{1}{c_m2\pi {d}_mL}{\int}_{a_1}^{a_2}\frac{da}{a}=\frac{\mathit{\ln}\left(\frac{a_2}{a_1}\right)}{c_m2\pi {d}_mL} $$

Where the myelin sheath extends from the radius a_1_ of the axon core to the outer radius a_2_, that is to the axon radius (See Fig. 10d, e).

Performing the (linear) cable theory, we obtain the diffusion equation:

12 $$ \frac{C_m}{L}\frac{\partial v}{\partial t}=\frac{\pi {a}_1^2}{r_L}\frac{\partial^2v}{\partial {x}^2} $$

The diffusion coefficient is:

13 $$ D=\frac{\pi {a}_1^2L}{C_m{r}_L}=\frac{a_1^2\mathit{\ln}\left(\frac{a_2}{a_1}\right)}{2{c}_m{r}_L{d}_m} $$

Where r_L_ is the intracellular resistivity. The optimal value of the internal radius a_1_ - which maximizes the diffusion constant- is *a*_1_~0.6*a*_2_ [8]. In the case of a myelinated axon the propagation velocity is thus proportional to the outer radius a_2_, that is to the axon radius, while for an unmyelinated axon it is proportional to the square root of the axon radius (a_2_) [8].

14 $$ \mathrm{v}\sim {\mathrm{a}}_2. $$

Let us show with an example the versatility of the particle description of nerve impulses. Here we exploit the physics of particle accelerators [39, 40] because from our point of view the functionality of a myelinated axon is that of a (micro) linear particle accelerator (μLINAC).

The myelinated regions behave like Faraday cages (drift tubes) while at the gaps of the nodes of Ranvier there is a non-zero electric field that provides the acceleration of the particle along the axon (see Fig. 10f). During the acceleration the velocity increases monotonically. In the ith drift tube the velocity v_i_ is reached. Now, considering the effective mass m _ñ_ of a nuon, we thus have an energy:

15 $$ {E}_i=\frac{1}{2}{m}_{\overset{\sim }{n}}{v}_i^2 $$

From cable theory we deduce that the average velocity is proportional to the radius of the myelin axon (14). In this way, we can estimate the effective mass and charge of the nuon and the modulus of the electric field at the nodes of Ranvier. This is a crucial result. To explain the biophysical mechanism of demyelinating pathologies we can use the nuon model because it provides advantages over the “classic” electrophysiological description. However, the model is *congruent* with the “classic” description because a spike is the electrophysiological trace and probe of the passage of a nuon.

Demyelination, due to the interruption of the paranodal myelin circuits, causes the dispersion of all the ion channels, pumps and exchangers along the axon [6, 31–36]. Sodium overload causes axonal calcium to reach toxic levels and so on [31]. As the conduction velocity in normal conditions (up to circa 150 m/s) is much higher than the velocity in pathological conditions (about 5 or 10 m/s), we can predict that, in pathological conditions, the resultant of the field-forces on the system will contain a finite set of deterministic dissipative fields acting on the demyelinated axon, generating instabilities and losses (that we can think as in the damaged drift tubes of our μLINAC). This model can also offer an operative tool characterized by self-similarity and reproducibility properties for polytype diffusion, since the etiology of the disease is presumably caused by a pathological (inflammatory) process that affects the whole body. Knowledge and measurement of these dissipative fields can therefore lead to significant progress in the study and treatment of neurodegenerative and demyelinating diseases. Furthermore, our considerations on the dissipative field model can be used to define a special circuitry intended to integrate the equivalent models (See Richardson [41]).

## Conclusions and possible insights

In this work we found a particle description of action potentials, based on considerations of statistical mechanics of complex and disordered systems, independently of classic electrophysiological models, such as Hodgkin-Huxley (HH).

Nevertheless, as soon as we consider the action potential as the electro-physiological trace of the nuon, we thus have the opportunity to exploit a full dualism of points of view and formal descriptions in order to describe the generation and propagation of nerve impulses, especially when classic electrophysiological models break down. In this case, SFT [ñ] is a powerful tool that allows us to use the techniques and results of theoretical and general physics [42, 43]. As we have just pointed out in the previous paragraph for the case of saltatory conduction, it is advantageous to exploit the dualism performing both the representations. But we expect the dual representation to be useful in many other cases as well. Functional Magnetic Resonance (*f*MRI) can be integrated by specific hardware devices and algorithms currently employed in particle physics in order to obtain real-time velocity field maps, even led by connectomes [44]. A detailed integrated real time imaging is therefore suitable to study a non-active area of the brain (i.e. in the presence of ischemia, injury, ictus, neurodegenerative pathology or tumor), by considering an” activity” tensor dependent on the nuon frequencies and fluxes defined on a dendritic density field [45]. Furthermore, the study of the activity tensor with the particle model can try to explain evolutionary puzzles related to multiple sclerosis, difficult to solve with electrophysiological models (see [46]). Other possible applications will exploit “nuon coding” [7] to study and develop complex networks, quantum computing, artificial intelligence, machine and deep learning, cryptography, ultra-fast lines for entanglement experiments and so on. A particle model of synaptic transmission through a “nuon number” conservation law can be also derived and will be the subject of a future work.

## Data Availability

Not applicable.

## Abbreviations

HH: Hodgkin-Huxley models; INM: Ising Neural Machine
SFT [n˜]: Statistical Field Theory of the Transmission of Nerve Impulses
*f* MRI: Functional Nuclear Magnetic Resonance

## Footnotes

1 The concept of *congruence* was used by the present author in the context of the unconventional statistical calculus system called “SHT”, based on the theory of categories and intended for the study of complex and disordered systems (derived and patented between 1997 and 2011 [16]). From a practical point of view, the SHT calculus does not “destructively interfere” with the sample, but analyzes the system *sic* *rebus stantibus*, considering also the “junk”, the environmental background and the noise. SHT treats the sample as a “dynamic system”. It studies maps and transforms, looks for critical points and transitions, bifurcations and attractors. For example, if SHT finds an attractor, it will become a “category” of the experiment. This is precisely our meaning of “congruence”. From a statistical point of view, a congruent model has the same properties as a probability density. Any metatheory is not a category of the experiment. In the case of very large and complex systems, the analysis is generally carried out on many logical levels (see for example some works on complex systems and particle physics [17, 18]). In other cases, the analysis is conducted by arranging the data on an REM energy landscape and studying the configurations of minimum entropy [19].

2 As a functional icon synonymous of complexity, we chose the “complex” polyhedron called Icosi-icosahedron first described by Edmund Hess in 1876. It is the result from the auto dual composition of 10 tetrahedra enclosing a dodecahedron, all intersected by an icosahedron. The compound of ten tetrahedra is one of the five regular polyhedral compounds. This polyhedron can be seen as either a stellation of the icosahedron or a compound. The vertices correspond to the pairs 2 {5,3} [10 {3,3}] 2 {3,5}. After “Polytopes and their Incident Matrices”, courtesy of Richard Klitzing [20, 21].
