# Correction to: Statistical field theory of the transmission of nerve impulses

**DOI:** 10.1186/s12976-021-00141-2

**Published:** 2021-03-05

**Authors:** Gianluigi Zangari del Balzo

**Affiliations:** Sapienza Università di Roma, Rome, Italy

**Correction to: Theor Biol Med Model (2021) 18:1**

**https://doi.org/10.1186/s12976-020-00132-9**

Following publication of the original article [1], we were notified that the equation (8) in the pdf was missing the right parenthesis of the Dirac ket “>”.

The original article has been corrected.
